# Examining Criteria for Defining Persistent Post-concussion Symptoms in Children and Adolescents

**DOI:** 10.3389/fneur.2021.614648

**Published:** 2021-02-23

**Authors:** Grant L. Iverson, Justin E. Karr, Bruce Maxwell, Ross Zafonte, Paul D. Berkner, Nathan E. Cook

**Affiliations:** ^1^Department of Physical Medicine and Rehabilitation, Harvard Medical School, Boston, MA, United States; ^2^MassGeneral Hospital for Children Sports Concussion Program, Boston, MA, United States; ^3^Spaulding Rehabilitation Hospital, Charlestown, MA, United States; ^4^Spaulding Research Institute, Charlestown, MA, United States; ^5^Department of Psychology, University of Kentucky, Lexington, KY, United States; ^6^Department of Computer Science, Colby College, Waterville, ME, United States; ^7^Home Base, A Red Sox Foundation and Massachusetts General Hospital Program, Department of Physical Medicine and Rehabilitation, Brigham and Women's Hospital, Boston, MA, United States; ^8^College of Osteopathic Medicine, University of New England, Biddeford, ME, United States

**Keywords:** mild traumatic brain injury, brain trauma, outcome research, postconcussional syndrome, pediatrics, assessment, symptoms

## Abstract

Researchers operationalize persistent post-concussion symptoms in children and adolescents using varied definitions. Many pre-existing conditions, personal characteristics, and current health issues can affect symptom endorsement rates in the absence of, or in combination with, a recent concussion, and the use of varied definitions can lead to differences in conclusions about persistent symptoms and recovery across studies. This study examined how endorsement rates varied by 14 different operational definitions of persistent post-concussion symptoms for uninjured boys and girls with and without pre-existing or current health problems. This cross-sectional study included a large sample (age range: 11–18) of girls (*n* = 21,923) and boys (*n* = 26,556) without a recent concussion who completed the Post-Concussion Symptom Scale at preseason baseline. Endorsements rates varied substantially by definition, health history, and current health issues. The most lenient definition (i.e., a single mild symptom) was endorsed by most participants (54.5% of boys/65.3% of girls). A large portion of participants with pre-existing mental health problems (42.7% of boys/51.5% of girls), current moderate psychological distress (70.9% of boys/72.4% of girls), and insufficient sleep prior to testing (33.4% of boys/47.6% of girls) endorsed symptoms consistent with mild ICD-10 postconcussional syndrome; whereas participants with no current or prior health problems rarely met this definition (1.6% of boys/1.6% of girls). The results illustrate the tremendous variability in the case definitions of persistent symptoms and the importance of harmonizing definitions across future studies.

## Introduction

The typical recovery time from a sport-related concussion in children and adolescents ranges from a few days to 1 month (1–7), but many youth have symptoms that persist for considerably longer than 1 month (8–13). However, the definition of “persistent symptoms” varies considerably across studies. Some studies define recovery as being completely asymptomatic or having complete symptom resolution (11, 13, 14) and having a score of zero on a symptom questionnaire (15–17), thus endorsing a single symptom during a post-injury evaluation meets the threshold for having persistent symptoms, not being recovered, or both. Another study allowed “a few” symptoms to be endorsed with very mild ratings of “1” or “2” on a 6-point scale as the definition of being recovered (18), while another allowed for either being asymptomatic, reporting that the athlete returned to pre-injury symptom status, or symptom improvement allowing for return-to-play (4). Other studies have attempted to compare retrospective ratings of pre-injury symptoms to post-injury symptoms. For example, one study considered endorsing a single symptom as “2” or greater, indicating that the symptom was considered worse compared to how the participant felt before the injury, as evidence of persistent symptoms (8). The large Canadian “5P” multi-center study defined persistent symptoms as having 3 or more symptoms endorsed as greater, to any degree, than their retrospective pre-injury symptom rating (9). Other studies examine symptom reports in combination with other clinical data, such as by requiring resolution of symptoms (19) or being symptom-free at rest and with exertion (17), in combination with results of balance testing, neurocognitive performances, and normal resumption of academic activities.

Persistent symptoms following concussion are sometimes referred to as the postconcussional syndrome. There are multiple challenges regarding the identification and classification of pediatric postconcussional syndrome, several sets of criteria have been proposed, and more research is needed to validate and refine criteria in children (20, 21). With that said, according to the International Classification of Diseases, 10th Revision (ICD-10) criteria for postconcussional syndrome, a person must have symptoms in at least 3 of 6 domains that persist for more than 1 month (22). Researchers applying those ICD-10 criteria in sport concussion studies with youth focus on having one or more symptoms in at least 3 of the 4 primary domains: physical, emotional, cognitive, and insomnia (23, 24). When the ICD-10 symptom criteria were applied to a sample of more than 31,000 *uninjured* adolescents undergoing preseason baseline evaluations, 19% of boys and 27% of girls endorsed sufficient symptoms, in the absence of concussion, to meet criteria for the diagnosis of postconcussional syndrome (23). Moreover, the rate of symptom endorsement was much greater in adolescents with attention-deficit/hyperactivity disorder (ADHD), learning disorders, a prior history of treatment for headaches, and a history of mental health problems (23, 24).

Concussion-like symptom reporting is relatively common in youth in their daily lives (23) and it is not known how common it is for youth to meet various definitions of “persistent symptoms” in the absence of recent concussion. To inform both research and clinical practice, definitions of persistent symptoms should be examined, stratified by gender, in *uninjured* youth with pre-existing conditions such as ADHD, learning disorders, and prior mental health problems, as well as youth who might be experiencing situational symptoms relating to psychological distress (25) or insufficient sleep (26, 27). The purpose of this study is to compare 14 different operational definitions for persistent symptoms in a sample of approximately 50,000 *uninjured* children and adolescents. We hypothesized that there would be a linear increase in the rates in which youth would meet these definitions, following a conceptually logical gradient of more restrictive to more permissive criteria. Moreover, we hypothesized that the rates would be much higher in (i) youth with pre-existing conditions, (ii) youth who reported getting insufficient sleep the night before completing the symptom questionnaire, and (iii) youth who reported experiencing psychological distress in the days leading up to the baseline preseason evaluation.

## Materials and Methods

### Participants

Participants were drawn from a sample of 50,018 children and adolescents, ages 11–18, from Maine, USA who denied have sustained a concussion in the past 6 months and completed the Immediate Post-Concussion Assessment and Cognitive Testing (ImPACT®) battery between 2009 and 2015 as part of a preseason baseline evaluation before participating in their athletic season. Participants were excluded if they completed ImPACT® in a language other than English (*n* = 802) or reported a history of treatment for epilepsy or seizures (*n* = 505), treatment for meningitis (*n* = 193), or brain surgery (*n* = 100). The final sample included 48,479 children and adolescents (age: *M* = 15·3, SD = 1·4, range: 11–18). There were 21,923 (45.2%) girls (age *M* = 15.2, SD = 1.4) and 26,556 (54.8%) boys (age: *M* = 15.4, SD = 1.5). Institutional review board approval to create and use this de-identified database was obtained from Colby College (primary) and from Spaulding Rehabilitation Hospital (secondary use).

### Measure

ImPACT® is a computerized assessment that includes demographic, academic, and health history questionnaires, the Post-Concussion Symptom Scale (PCSS), and a series of neurocognitive tests. The academic and health history questionnaires ask participants to report (i.e., yes or no) whether they had previous special education services, repeated an academic grade, received speech therapy; diagnoses of ADHD, autism, or learning disability or dyslexia; or previous treatment for headaches, migraines, epilepsy/seizures, brain surgery, meningitis, substance/alcohol use, or a psychiatric condition (e.g., anxiety or depression). Participants are also asked to report the number of previously diagnosed concussions they have experienced. The PCSS is a 22-item self-report questionnaire. Participants rate the severity at which they are currently experiencing physical, cognitive, and emotional symptoms on a 0–6 Likert-type scale (i.e., 0 = Not present, 1–2 = Mild, 3–4 = Moderate, and 5–6 = Severe).

### Statistical Analyses

We calculated the frequencies at which uninjured children and adolescents endorsed symptoms consistent with different operational definitions for persistent post-concussion symptoms. These definitions are provided in Table 1. The definitions varied in the number of symptoms required (i.e., ranging from a minimum of 1–3 symptoms endorsed) and the severity at which symptoms needed to be rated to be considered endorsed (i.e., 1 or greater for a mild symptom and 3 or greater for a moderate symptom). They also varied by the number of symptoms considered in the definitions. Some considered all 22 symptoms from the PCSS, whereas others considered just a subset of 16 symptoms consistent with ICD-10 postconcussional syndrome symptom domains of physical (i.e., headache, nausea, balance problems, dizziness, fatigue, sensitivity to light, and sensitivity to noise), emotional (i.e., irritability, nervousness, sadness, feeling more emotional), cognitive (i.e., feeling mentally foggy, difficulty concentrating, difficulty remembering), and insomnia/sleep-related symptoms (i.e., trouble falling asleep, sleeping less than usual). Two definitions were based on the ICD-10 symptom criteria for postconcussional syndrome. We created more and less stringent versions. A rating of “mild” severity (i.e., item rating of 1 or greater on the Likert scale) for at least one symptom in at least three of the four domains would qualify a participant as meeting criteria for mild ICD-10 postconcussional syndrome and a rating of 3 or greater for at least one symptom in at least three domains would qualify a participant as meeting criteria for moderate ICD-10 postconcussional syndrome.

**Table 1:** Operational definitions for having symptoms.

1. Endorsing 1 or more symptoms out of 22 as “mild” or greater (i.e., “1” or greater)
2. Endorsing 1 or more symptoms out of 16 as “mild” or greater (i.e., “1” or greater)
3. Endorsing 1 or more symptoms out of 22 as “moderate” or greater (i.e., “3” or greater)
4. Endorsing 1 or more symptoms out of 16 as “moderate” or greater (i.e., “3” or greater)
5. Endorsing 2 or more symptoms out of 22 as “mild” or greater (i.e., “1” or greater)
6. Endorsing 2 or more symptoms out of 16 as “mild” or greater (i.e., “1” or greater)
7. Endorsing 2 or more symptoms out of 22 as “moderate” or greater (i.e., “3” or greater)
8. Endorsing 2 or more symptoms out of 16 as “moderate” or greater (i.e., “3” or greater)
9. Endorsing 3 or more symptoms out of 22 as “mild” or greater (i.e., “1” or greater)
10. Endorsing 3 or more symptoms out of 16 as “mild” or greater (i.e., “1” or greater)
11. Endorsing 3 or more ICD-10 PCS symptom categories as “mild” or greater (i.e., “1” or greater)
12. Endorsing 3 or more symptoms out of 22 as “moderate” or greater (i.e., “3” or greater)
13. Endorsing 3 or more symptoms out of 16 as “moderate” or greater (i.e., “3” or greater)
14. Endorsing 3 or more ICD-10 PCS symptom categories as “moderate” or greater (i.e., “3” or greater)

*ICD-10 PCS = Postconcussional syndrome based on the International Classification of Diseases, Tenth Edition. Definitions using 22 symptoms considered all symptoms from the Post-Concussion Symptom Scale (PCSS). Definitions using 16 symptoms considered just 16 symptoms from the PCSS consistent with ICD-10 PCS symptom categories of physical (i.e., headache, nausea, balance problems, dizziness, fatigue, sensitivity to light, and sensitivity to noise), emotional (i.e., irritability, nervousness, sadness, feeling more emotional), cognitive (i.e., feeling mentally foggy, difficulty concentrating, difficulty remembering), and insomnia/sleep-related (i.e., trouble falling asleep, sleeping less than usual). A rating of “mild” severity (i.e., item rating of 1 or greater on the Likert scale) for at least one symptom in at least three of the four categories would qualify a participant as meeting criteria for mild ICD-10 PCS and a rating of 3 or greater for at least one symptom in at least three categories would qualify a participant as meeting criteria for moderate ICD-10 PCS*.

Student athletes were grouped based on health and academic history, current health issues, and combinations thereof. We examined a total of 28 subgroups. Current health issues included insufficient sleep, headaches, and psychological distress. Sleep insufficiency was defined as reporting 6 or fewer hours of sleep the night before completing the symptom questionnaire. Current mild and moderate psychological distress was defined as endorsing one or more of the four PCSS emotional symptoms (i.e., irritability, nervousness, sadness, feeling more emotional) as “mild' (1) or greater or “moderate” (3) or greater, respectively. Current mild and moderate headaches were defined as endorsing headaches as “mild” (1) or greater or “moderate” (3) or greater on the PCSS, respectively. We calculated the rate at which participants met criteria for each operational definition of persistent symptoms for the total sample and each category separately for boys and girls. Two subgroups were formed composed of student athletes approximating a currently healthy and typically developing sample: the total sample with no pre-existing conditions (i.e., responded no to all academic and health history questions) and the total sample with no pre-existing conditions and no current health issues (i.e., responded no to all academic and health history questions, reported >6 h of sleep the night before the assessment, and did not endorse current headaches or any emotional symptoms). The other subgroups were composed of youth with different health conditions and current health issues: learning disability, ADHD, ADHD and learning disability, ADHD and insufficient sleep, ADHD and mild current psychological distress, ADHD and moderate current psychological distress, mild current headaches, moderate current headaches, past treatment for headaches, past treatment for headaches and mild current headaches, past treatment for headaches and moderate current headaches, insufficient sleep, mild current psychological distress, moderate current psychological distress, past treatment for a psychiatric condition, past treatment for a psychiatric condition and insufficient sleep, past treatment for a psychiatric condition and mild current psychological distress, past treatment for a psychiatric condition and moderate current psychological distress, no prior concussions, and different numbers of prior concussions (i.e., ranging from 1 to 4 or greater).

## Results

**Figure 1:**
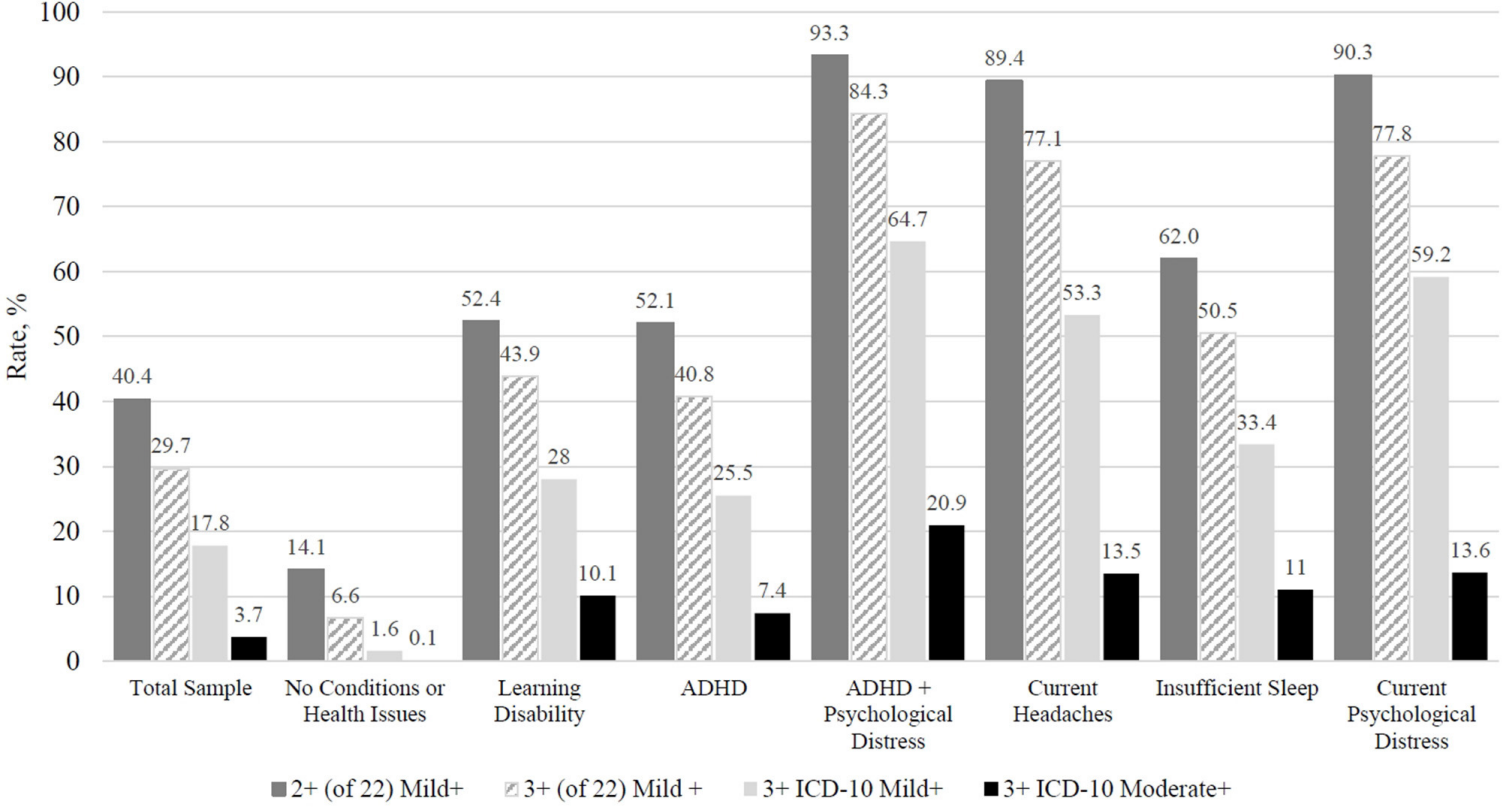
Comparing rates of boys, with no concussion in the past 6 months, endorsing symptoms consistent with 4 different operational definitions for persistent symptom reporting (percentages). ADHD, Attention-deficit/hyperactivity disorder; ICD-10 Mild+, Post-concussional syndrome of mild or greater severity based on the International Classification of Diseases, Tenth Edition; ICD-10 Moderate+, Post-concussional syndrome of moderate or greater severity based on the International Classification of Diseases, Tenth Edition. Total Sample = 26,556, No Conditions or Health Issues = 10,205, Learning Disability = 1,333, ADHD = 3,593, ADHD + Psychological Distress = 1,184, Current Headaches = 4,034, Insufficient Sleep (6 or fewer hours the night before) = 1,994, Current Psychological Distress (a rating of “1” or greater on at least one emotional symptom) = 6,647. Criteria: Endorsing two or more symptoms out of 22 as “mild” or greater (i.e., “1” or greater). Endorsing three or more symptoms out of 22 as “mild” or greater (i.e., “1” or greater). Endorsing three or more ICD-10 PCS symptom categories as “mild” or greater (i.e., “1” or greater). Endorsing three or more ICD-10 PCS symptom categories as “moderate” or greater (i.e., “3” or greater).

**Figure 2:**
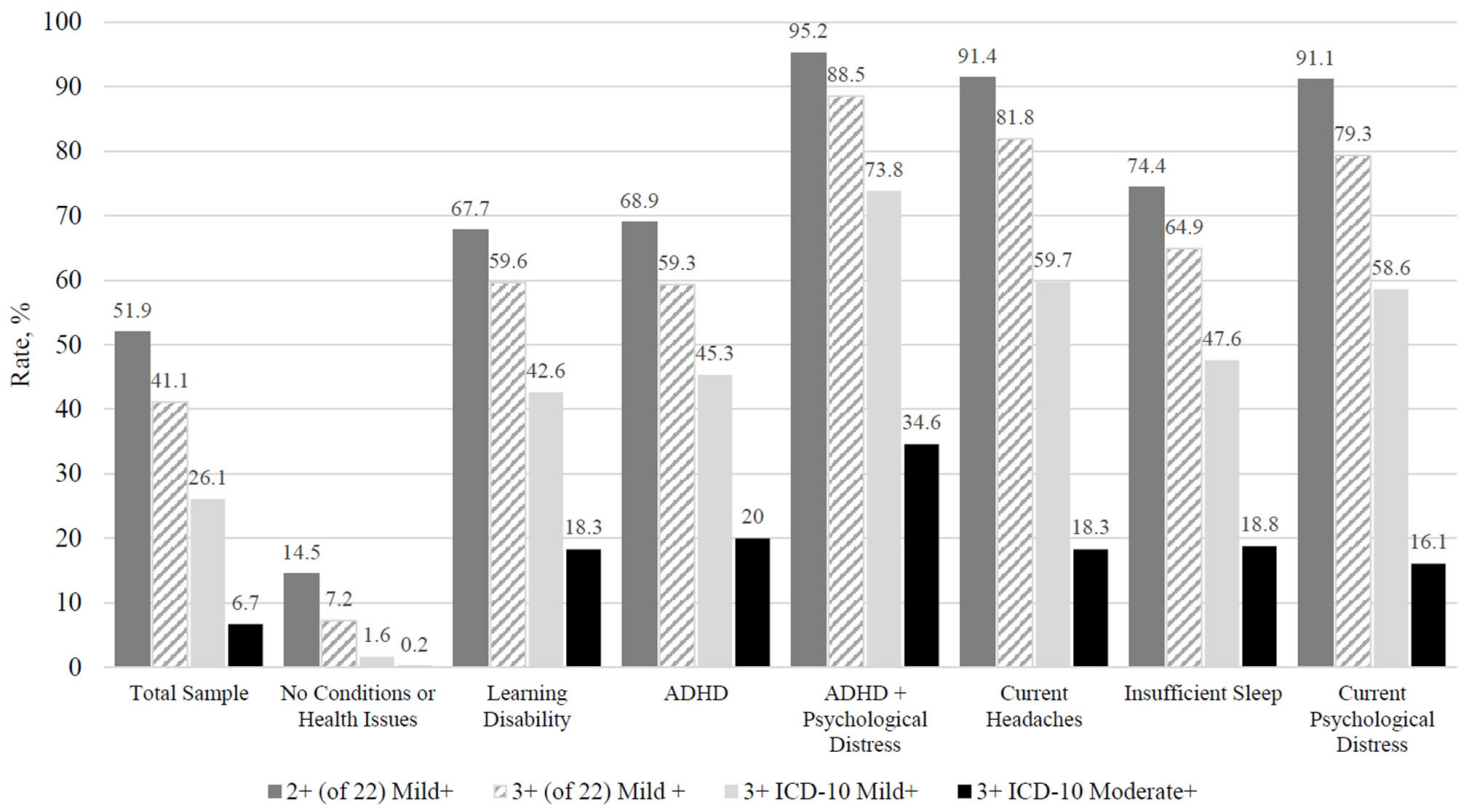
Comparing rates of girls, with no concussion in the past 6 months, endorsing consistent with 4 different operational definitions for persistent symptom reporting (percentages). ADHD, Attention-deficit/hyperactivity disorder; ICD-10 Mild+, Post-concussional syndrome of mild or greater severity based on the International Classification of Diseases, Tenth Edition; ICD-10 Moderate+, Post-concussional syndrome of moderate or greater severity based on the International Classification of Diseases, Tenth Edition. Total Sample = 21,923, No Conditions or Health Issues = 7,751, Learning Disability = 802, ADHD = 1,422, ADHD + Psychological Distress = 790, Current Headaches = 5,177, Insufficient Sleep (6 or fewer hours the night before) = 1,821, Current Psychological Distress (a rating of “1” or greater on at least one emotional symptom) = 8,808. Criteria: Endorsing two or more symptoms out of 22 as “mild” or greater (i.e., “1” or greater). Endorsing three or more symptoms out of 22 as “mild” or greater (i.e., “1” or greater). Endorsing three or more ICD-10 PCS symptom categories as “mild” or greater (i.e., “1” or greater). Endorsing three or more ICD-10 PCS symptom categories as “moderate” or greater (i.e., “3” or greater).

The rates at which boys and girls with different pre-existing conditions and current health issues endorsed symptoms consistent with 14 definitions of persistent symptoms are reported in Tables 2, 3, respectively. Rates for a select number of groups and definitions are presented visually in Figures 1, 2. The most lenient operational definition for persistent symptoms was a single symptom on the PCSS, which was endorsed at the highest frequency for all participant categories. All participants endorsing current headaches or psychological distress by default met criteria for this definition, because an endorsement of headaches or emotional symptoms led to a score of at least 1 on the PCSS. Among youth with insufficient sleep the night before completing the symptoms questionnaire, 4 of 5 girls (82.5%) and 3 of 4 boys (73.5%) met this definition for persistent symptoms.

**Table 2:** Percentages of boys endorsing symptoms based on each operational definition.

		**Operational definitions of endorsing symptoms (see** **Table 1****)**													
**Group**	**N**	**1**	**2**	**3**	**4**	**5**	**6**	**7**	**8**	**9**	**10**	**11**	**12**	**13**	**14**
Total sample	26,556	54.5%	52.1%	23.7%	22.1%	40.4%	36.7%	12.9%	11.3%	29.7%	25.8%	17.8%	7.5%	6.1%	3.7%
Total sample, no pre-existing conditions	14,667	48.0%	45.5%	17.5%	16.0%	33.9%	30.3%	8.3%	7.1%	23.5%	20.0%	13.4%	4.2%	3.3%	1.9%
Total sample, no conditions, no current health issues	10,205	27.7%	24.2%	7.0%	5.8%	14.1%	10.7%	2.0%	1.5%	6.6%	4.3%	1.6%	0.5%	0.3%	0.1%
Ages 11-12, no pre-existing conditions	858	35.5%	32.6%	10.4%	9.3%	21.4%	20.2%	4.5%	3.8%	15.5%	13.3%	8.6%	2.1%	1.4%	0.8%
Ages 13-15, no pre-existing conditions	9,338	48.3%	45.4%	16.5%	15.0%	33.6%	29.7%	7.6%	6.3%	22.9%	19.2%	13.0%	3.8%	3.0%	1.8%
Ages 16-18, no pre-existing conditions	4,471	50.0%	48.2%	20.8%	19.4%	36.7%	33.5%	10.4%	9.5%	26.2%	23.0%	15.3%	5.3%	4.4%	2.3%
Learning disability	1,333	65.4%	63.8%	38.7%	37.4%	52.4%	49.4%	26.4%	24.2%	43.9%	39.5%	28.0%	17.6%	15.1%	10.1%
ADHD	3,593	65.5%	63.2%	35.6%	33.7%	52.1%	48.1%	21.8%	19.4%	40.8%	36.2%	25.5%	13.4%	11.2%	7.4%
ADHD and learning disability	642	69.2%	67.6%	43.5%	41.9%	57.0%	53.9%	31.2%	28.8%	49.4%	44.5%	32.1%	21.5%	18.8%	12.9%
ADHD and insufficient sleep	379	77.6%	76.3%	52.5%	50.9%	66.2%	63.3%	38.8%	36.7%	55.1%	51.7%	37.5%	26.1%	23.2%	15.6%
ADHD and mild current psychological distress	1,184	100%	100%	66.3%	64.5%	93.3%	91.9%	47.7%	43.8%	84.3%	79.6%	64.7%	33.1%	29.1%	20.9%
ADHD and moderate current psychological distress	476	100%	100%	100%	100%	96.8%	95.8%	82.4%	78.4%	91.4%	88.4%	72.9%	64.1%	59.9%	47.5%
Mild current headaches	4,034	100%	100%	55.7%	53.5%	89.4%	86.7%	35.7%	32.4%	77.1%	72.1%	53.3%	24.3%	20.7%	13.5%
Moderate current headaches	980	100%	100%	100%	100%	94.2%	92.8%	73.4%	70.1%	86.3%	82.7%	63.6%	55.8%	51.0%	32.7%
Past treatment for headaches	2,258	72.0%	70.0%	43.4%	40.9%	60.3%	56.1%	27.8%	24.9%	49.6%	44.2%	32.9%	19.0%	16.1%	10.2%
Past treatment for headaches and mild current headaches	745	100%	100%	70.3%	68.6%	92.5%	90.1%	48.5%	44.7%	82.1%	76.8%	59.5%	36.8%	31.5%	20.4%
Past treatment for headaches and moderate current headaches	299	100%	100%	100%	100%	94.6%	93.3%	75.3%	71.2%	88.0%	83.9%	66.2%	59.9%	52.8%	34.1%
Insufficient sleep	1,994	73.5%	72.0%	46.3%	44.9%	62.0%	58.4%	31.2%	29.2%	50.5%	45.9%	33.4%	20.3%	17.6%	11.0%
Mild current psychological distress	6,647	100%	100%	54.6%	52.6%	90.3%	88.0%	35.7%	32.3%	77.8%	72.9%	59.2%	23.4%	20.0%	13.6%
Moderate current psychological distress	2,025	100%	100%	100%	100%	95.1%	93.8%	76.7%	73.5%	87.4%	84.9%	70.9%	57.5%	52.3%	39.8%
Past treatment for a psychiatric condition	1,290	80.9%	79.1%	52.4%	50.5%	71.5%	68.1%	37.4%	34.1%	61.0%	56.3%	42.7%	27.6%	23.2%	15.9%
Past treatment for a psychiatric condition and insufficient sleep	172	89.5%	89.5%	73.3%	71.5%	84.3%	84.3%	59.9%	57.6%	75.0%	74.4%	58.7%	45.9%	41.3%	30.2%
Past treatment for a psychiatric condition and mild current psychological distress	735	100%	100%	73.1%	71.3%	94.1%	92.8%	55.6%	51.7%	87.1%	83.8%	69.0%	43.4%	37.7%	26.9%
Past treatment for a psychiatric condition and moderate current psychological distress	379	100%	100%	100%	100%	96.3%	95.3%	83.6%	81.5%	92.1%	91.3%	76.5%	71.8%	66.0%	49.6%
No prior concussions	21,467	53.5%	51.0%	22.3%	20.7%	39.4%	35.6%	12.0%	10.4%	28.7%	24.9%	17.1%	6.8%	5.5%	3.3%
1 Prior concussion	3,179	58.4%	56.2%	27.3%	25.7%	43.0%	39.5%	14.9%	13.1%	32.6%	27.7%	19.1%	8.8%	7.3%	4.1%
2 Prior concussions	943	63.6%	61.8%	36.5%	33.9%	52.0%	47.9%	21.2%	19.2%	39.8%	36.4%	25.5%	14.2%	12.0%	7.5%
3 Prior concussions	305	66.2%	64.9%	41.6%	39.3%	53.4%	48.2%	25.9%	22.3%	40.0%	35.7%	27.9%	15.7%	14.4%	9.2%
4 or More prior concussions	175	65.1%	63.4%	46.3%	42.3%	57.1%	53.7%	31.4%	28.6%	46.3%	40.6%	30.9%	22.3%	20.0%	14.9%

*ADHD, Attention-deficit/hyperactivity disorder. The 14 operational definitions of having symptoms are provided in Table 1. Current health issues include insufficient sleep, mild headaches, and mild psychological distress. Sleep insufficiency is defined as reporting sleeping 6 or fewer hours the night before baseline testing. Mild current psychological distress is defined as endorsing one or more of the 4 emotional symptoms as “mild” (1) or greater. Moderate current psychological distress is defined as endorsing one or more of the 4 emotional symptoms as “moderate” (3) or greater*.

**Table 3:** Percentages of girls endorsing symptoms based on each operational definition.

		**Operational definitions of endorsing symptoms (see Table 1)**													
**Group**	**N**	**1**	**2**	**3**	**4**	**5**	**6**	**7**	**8**	**9**	**10**	**11**	**12**	**13**	**14**
Total Sample	21,923	65.3%	63.7%	31.3%	30.0%	51.9%	49.1%	19.2%	17.8%	41.1%	37.5%	26.1%	12.6%	11.2%	6.7%
Total Sample, No Pre-Existing Conditions	14,054	59.6%	57.8%	23.7%	22.5%	44.9%	42.2%	12.9%	11.7%	33.8%	30.4%	20.0%	7.6%	6.5%	3.6%
Total Sample, No Conditions, No Current Health Issues	7,751	29.6%	26.5%	7.0%	6.1%	14.5%	11.7%	2.1%	1.7%	7.2%	4.9%	1.6%	0.6%	0.5%	0.2%
Ages 11-12, No Pre-Existing Conditions	741	40.5%	39.3%	14.0%	13.8%	25.8%	24.3%	7.6%	6.9%	19.6%	18.4%	11.1%	4.5%	4.0%	2.0%
Ages 13-15, No Pre-Existing Conditions	9,840	60.2%	58.2%	22.6%	21.3%	44.9%	42.0%	12.0%	10.9%	33.3%	29.7%	19.6%	6.9%	6.0%	3.2%
Ages 16-18, No Pre-Existing Conditions	3,473	62.2%	60.6%	29.0%	27.8%	49.3%	46.6%	16.5%	15.0%	38.4%	34.9%	23.0%	10.0%	8.6%	4.9%
Learning Disability	802	75.9%	74.6%	50.0%	47.6%	67.7%	65.3%	35.4%	33.5%	59.6%	55.7%	42.6%	27.1%	24.8%	18.3%
ADHD	1,422	78.4%	77.8%	52.9%	51.6%	68.9%	66.8%	37.9%	35.9%	59.3%	55.5%	45.3%	29.6%	26.8%	20.0%
ADHD and Learning Disability	328	82.6%	81.7%	61.0%	59.5%	76.5%	74.7%	46.6%	44.5%	70.1%	66.2%	55.2%	38.4%	35.1%	27.7%
ADHD and Insufficient Sleep	179	90.5%	90.5%	77.7%	77.1%	87.2%	85.5%	62.0%	59.8%	79.3%	78.2%	66.5%	52.5%	48.6%	40.2%
ADHD and Mild Current Psychological Distress	790	100%	100%	75.2%	74.3%	95.2%	94.7%	60.4%	58.4%	88.5%	84.9%	73.8%	49.1%	44.9%	34.6%
ADHD and Moderate Current Psychological Distress	438	100%	100%	100%	100%	97.7%	97.5%	88.4%	86.8%	93.8%	92.5%	80.8%	75.8%	71.7%	57.3%
Mild Current Headaches	5,177	100%	100%	60.4%	59.2%	91.4%	89.9%	42.5%	40.0%	81.8%	78.4%	59.7%	30.8%	27.8%	18.3%
Moderate Current Headaches	1,496	100%	100%	100%	100%	95.3%	94.1%	76.6%	73.9%	90.1%	87.8%	70.5%	60.6%	56.1%	38.5%
Past Treatment for Headaches	2,164	82.7%	81.7%	54.8%	53.3%	72.5%	69.7%	38.4%	36.3%	62.5%	58.5%	43.3%	27.7%	24.7%	15.7%
Past Treatment for Headaches and Mild Current Headaches	1,065	100%	100%	74.0%	72.6%	92.3%	90.0%	54.5%	51.9%	84.7%	81.4%	63.8%	41.7%	37.7%	25.2%
Past Treatment for Headaches and Moderate Current Headaches	492	100%	100%	100%	100%	94.5%	92.9%	79.5%	76.8%	89.8%	87.8%	71.7%	64.4%	59.1%	40.2%
Insufficient Sleep	1,821	82.5%	81.7%	59.5%	58.5%	74.4%	72.1%	44.3%	42.1%	64.9%	61.6%	47.6%	31.5%	28.9%	18.8%
Mild Current Psychological Distress	8,808	100%	100%	57.0%	55.5%	91.1%	89.4%	39.6%	37.4%	79.3%	75.5%	58.6%	28.0%	25.3%	16.1%
Moderate Current Psychological Distress	3,225	100%	100%	100%	100%	96.4%	95.8%	81.1%	79.3%	91.0%	89.2%	72.4%	63.8%	59.9%	40.6%
Past Treatment for a Psychiatric Condition	1,819	86.1%	85.2%	61.5%	60.3%	78.2%	76.2%	49.3%	47.3%	70.5%	67.7%	51.5%	39.2%	36.8%	24.6%
Past Treatment for a Psychiatric Condition and Insufficient Sleep	273	94.9%	94.5%	83.2%	82.8%	91.6%	90.1%	73.6%	71.8%	86.4%	83.5%	69.6%	62.3%	60.1%	44.0%
Past Treatment for a Psychiatric Condition and Mild Current Psychological Distress	1,288	100%	100%	77.6%	76.7%	96.0%	95.1%	65.8%	63.4%	90.3%	88.0%	69.8%	53.3%	50.2%	34.1%
Past Treatment for a Psychiatric Condition and Moderate Current Psychological Distress	800	100%	100%	100%	100%	97.4%	96.8%	89.9%	88.1%	94.6%	93.8%	78.0%	77.8%	74.5%	52.9%
No Prior Concussions	19,025	64.4%	62.7%	30.0%	28.7%	50.7%	48.0%	18.1%	16.7%	39.9%	36.3%	25.1%	11.8%	10.4%	6.2%
1 Prior Concussion	1,936	71.9%	70.7%	39.6%	37.8%	59.3%	55.6%	25.4%	23.7%	48.2%	44.2%	31.6%	16.8%	14.9%	9.3%
2 Prior Concussions	410	74.1%	72.7%	47.3%	46.3%	62.4%	59.8%	32.7%	32.0%	55.1%	52.0%	37.6%	23.7%	21.5%	14.1%
3 Prior Concussions	142	83.8%	82.4%	56.3%	56.3%	75.4%	73.2%	40.8%	36.6%	64.8%	60.6%	41.5%	30.3%	27.5%	18.3%
4 or More Prior Concussions	59	84.7%	84.7%	67.8%	66.1%	72.9%	71.2%	47.5%	44.1%	64.4%	62.7%	52.5%	30.5%	28.8%	23.7%

*ADHD, Attention-deficit/hyperactivity disorder. The 14 operational definitions of having symptoms are provided in Table 1. Current health issues include insufficient sleep, headaches, and psychological distress. Sleep insufficiency is defined as reporting sleeping 6 or fewer hours the night before baseline testing. Current mild and moderate psychological distress was defined as endorsing one or more of the four emotional symptoms (i.e., irritability, nervousness, sadness, feeling more emotional) as “mild' (1) or greater or “moderate” (3) or greater, respectively*.

Rates of endorsement decreased when fewer symptoms were considered in the definition (i.e., 16 symptoms rather than 22 symptoms) and when moderate symptoms (i.e., rated as 3 or greater), rather than mild symptoms (i.e., rated as 1 or greater), were required by the definition. For example, 65.3% of girls met the definition when requiring 1 or more mild symptoms on the PCSS (definition 1), 51.9% of girls met the definition when requiring 2 or more mild symptoms (definition 5), and 41.1% of girls met the definition when requiring 3 or more mild symptoms (definition 9). Also, 40.4% of boys met the definition when requiring 2 or more symptoms rated mild or greater on the PCSS (definition 5) compared to just 12.9% when requiring 2 or more symptoms rated moderate or greater (definition 7). The moderate ICD-10 postconcussional syndrome definition (definition 14) was the least commonly met definition across groups, with rates for boys ranging from 49.6% among those with past psychiatric treatment and moderate current psychological distress to 0.1% among those with no pre-existing conditions or current health issues and rates for girls ranging from 57.3% among those with ADHD and moderate current psychological distress to 0.2% among those with no pre-existing conditions or current health issues.

Current psychological distress was strongly associated with the rates at which children and adolescents met these definitions for persistent symptoms. For instance, it was very uncommon for girls (2.1%) and boys (2.0%) without pre-existing conditions or current health issues to endorse 2 or more symptoms as moderate or greater (definition 7), but this definition was commonly met by girls (39.6%) and boys (35.7%) who were experiencing even mild current distress and very common among girls (81.1%) and boys (76.7%) experiencing moderate current distress. Insufficient sleep the night before completing the symptom questionnaire also had a notable impact. Specifically, it was very uncommon for girls (0.6%) and boys (0.5%) without pre-existing conditions or current health issues to endorse 3 or more symptoms as moderate or greater (definition 12), but this definition was much more frequently met by girls (31.5%) and boys (20.3%) who reported insufficient sleep the night before completing the symptom questionnaire.

Age was also associated with the rates at which children and adolescents met these definitions for persistent symptoms. Among students without pre-existing conditions, younger students (ages 11–12 years) had the lowest rates across all definitions. For example, about 1 of 3 boys (35.5%) and 2 of 5 girls (40.5%) ages 11–12 met the definition when requiring 1 or more mild symptoms on the PCSS (definition 1), compared to 1 of 2 older boys (48.3% ages 13–15 and 50.0% ages 16–18) and 3 of 5 older girls (60.2% ages 13–15 and 62.2% ages 16–18). Comparable proportions of students ages 13–15 and 16–18 met many of the definitions. However, for certain definitions there was a modest stair-step age effect such that students ages 11–12 had the lowest rate, followed by the 13–15-year age group, and the 16–18-year olds had the highest rate. For instance, for definition 7, students ages 11–12 had the lowest rate (4.5% of boys and 7.6% of girls), followed by the 13–15-year olds (7.6% of boys; 12.0% of girls), with the 16–18-year olds having the highest rate (10.4% of boys and 16.5% of girls).

## Discussion

We examined 14 different operational definitions of having persistent symptoms following a sport-related concussion in a large sample of uninjured children and adolescents. As hypothesized, a large percentage of children and adolescents, who had not sustained a recent concussion, endorsed sufficient symptoms to meet most of these definitions, and those with (i) pre-existing conditions, (ii) recent psychological distress, or (iii) insufficient sleep the night before completing the symptom questionnaire endorsed high rates of symptoms. It was quite common for uninjured children and adolescents to endorse at least one symptom (definition 1). In the total sample, 54.5% of boys and 65.3% of girls met this definition. Even when requiring at least 2 symptoms to be endorsed, 40.4% of uninjured boys and 51.2% of uninjured girls met this definition. Moreover, consistent with prior literature (23), even when requiring multiple symptoms to be endorsed across distinct domains (i.e., consistent with ICD-10 symptom criteria for postconcussional syndrome), roughly 1 in 5 uninjured boys (17.8%) and 1 in 4 uninjured girls (26.1%) met this definition. These results highlight the potential difficulty determining persistent post-concussion symptoms because substantial proportions of uninjured children and adolescents met many of the definitions. Furthermore, situational and contextual factors, such as emotional state and amount of sleep around the time a student completes their symptom questionnaire, can exert a strong effect on the rates at which children and adolescents met these definitions for persistent symptoms. These findings argue persuasively that we, as clinicians and researchers, are impelled to think about preexisting health conditions and insufficient sleep when interpreting concussion-like symptom reporting in the context of diagnosis and return to activity clearance.

### Clinical Implications

There are subgroups of children and adolescents who are highly likely to endorse “concussion-like” symptoms even if they have recovered from their injury. Symptom reporting in children and adolescents can be temperamental, situational, or both. That is, some youth experience, and are willing to endorse on a questionnaire, mild physical, cognitive, or emotional symptoms in their daily lives as part of their personality or dispositional temperament, and these symptoms are reasonably stable but can fluctuate. Youth can also experience (and endorse) these symptoms as a result of situational life stressors (25), insufficient sleep (26, 27), or both. Clinicians treating children and adolescents who sustained a concussion and may be experiencing a prolonged recovery are encouraged to carefully consider the potential contribution of temperamental and situational factors that might be related to symptom reporting.

As more time passes since the injury it can become increasingly challenging and complicated to interpret symptom reporting among children and adolescents. This is especially true for youth with co-occurring health conditions and current health concerns, such as psychological distress or insufficient sleep, that may be distinct from the concussion and related to other life circumstances or psychosocial factors. Moreover, when evaluating some children with persistent symptoms long after a concussion, there is potential for an over-emphasis on, or misattribution of, preexisting symptoms or life stress related symptoms, to the concussion, which might contribute to a nocebo effect (28).

Age was also related to symptom reporting such that younger students (ages 11–12 years) had the lowest rates of symptom endorsement across all definitions. This age effect was examined among students without pre-existing conditions and it will also be important for future studies to examine potential interactions among factors influencing symptom reporting, such as between age and pre-existing conditions.

Clinicians working in concussion clinics will also recognize another social psychological phenomenon relating to the doctor-patient relationship. That is, some youth begin to realize, after attending several appointments, that the post-concussion symptom questionnaire is being used to document lingering “concussion symptoms,” not just “symptoms,” and thus the child might mark “zero” to convey the impression that he or she has recovered from the concussion, even if a mild degree of that symptom is present. Therefore, responding “zero” *after* an injury might mean something *different* than responding “zero” *before* an injury, during baseline preseason testing. A zero symptom rating after an injury might reflect: (i) no experience of that symptom, (ii) a mild experience of the symptom but the youth attributes the cause to something other than concussion, (iii) a denial of a symptom that is actually present due to a limited understanding of what that symptom is or limited personal insight into the symptom, or (iv) a desire to convince the doctor that he or she has recovered from the concussion despite lingering symptoms (and those symptoms may or may not actually be related to the concussion). Other reasons may explain why athletes do or do not endorse symptoms, such as secondary reactions, which include stress and somatic symptoms related to a strong desire for return-to-play, prohibition from playing a sport they greatly enjoy, social pressure from teammates while being held from play, concerns about missing tryouts or critical games, falling behind in school, and parental concerns about their injury (2).

### Limitations

This study has several important limitations. The health history variables were determined via self-report and we were unable to verify this information through parent corroboration or medical chart review; however, self-report is not necessarily unreliable. Adolescents who undergo multiple baseline assessments, on average 2 years apart, are highly consistent in reporting their concussion history and a personal history of ADHD (29). Further, it is common in large data collection efforts in sport concussion research to determine health history through self-report. Another limitation is the criteria used for defining the postconcussional syndrome, for which we applied the ICD-10 criteria. Although the ICD-10 is an established diagnostic manual, there are alternative sets of proposed criteria for pediatric post-concussion syndrome and for defining persistent symptoms in the literature (20) that, if used, may have yielded different results. Data collection only involved the ImPACT® battery, meaning additional scales for quantifying current health problems (e.g., psychological distress, sleep problems, and headache severity) were not administered. Insufficient sleep was defined based on self-reported sleep duration the night prior to the assessment and may or may not reflect more chronic sleep issues. Psychological distress and headaches were defined based on PCSS item endorsement. This reliance on the PCSS to operationalize current health problems was circular; meaning if any health problem was endorsed, as a default, certain definitions of persistent symptoms were also endorsed. These rates may have differed if separate instruments were administered to query current psychological health, sleep quality, and headache severity. We had limited demographic information about the students and did not have, for example, students' race, ethnicity, or socioeconomic status. Understanding potential sociocultural differences in symptom reporting represents an important future research priority.

### Directions for Future Research

The study findings expose many challenges that future researchers need to address through new investigations and improved consistency in methodology. The results suggest that the potential for false positive categorizations of participants as having persistent post-concussion symptoms can be quite high in research on adolescent student athletes, especially when applying rather lenient definitions for persistent symptoms (e.g., 1 or 2 mild symptoms endorsed) and when youth present with preexisting health conditions and/or insufficient sleep. Moreover, the potential rate of false positives will vary according to how stringent or lenient a given definition for “persistent” symptoms is. Further, youth with pre-existing health conditions and current health issues endorse symptoms that may or may not be typically associated with their pre-existing condition or health issue. For example, youth athletes with ADHD endorse a wide variety of concussion-like symptoms on baseline assessments, not just symptoms typically attributable to their ADHD (e.g., difficulty concentrating) (24, 30). Also, insufficient sleep is associated with a diverse array of concussion-like symptoms, not just the sleep-related symptoms (26). In certain types of studies, it might be possible to track symptom endorsement over time and consider change or stability among symptom ratings in the context of youths' treatment and rehabilitation progress, such as reports of improvement from their physical therapist, or input from caregivers regarding their observations of change or continued difficulties in daily life.

Rates of symptom endorsement may vary by setting, and future studies could apply the definitions reported herein, and new definitions, to symptom reporting in different environments, such as primary care offices or specialty clinics. New definitions for persistent symptoms may be based on those used in clinical practice, which could involve additional methods aside from self-report symptom questionnaires, such as parent-report, clinical interview, and/or the use of other clinical tests, such as neurocognitive, balance, visual, or vestibular tests. Lastly, future work should propose and seek to validate standardized criteria that harmonize how persistent symptoms are defined across studies. The present study informs those efforts but is insufficient for making recommendations regarding which definitions are most appropriate, because this study involves only symptom reporting before an injury, not an analysis of youth with persistent symptoms. Variation in how researchers and clinicians characterize “persistent symptoms” following concussion presents obvious challenges to advancing our knowledge of persistent post-concussion symptoms in youth.

## Data Availability Statement

Statistical analyses and outputs of all results used in this article will be made available by the authors, without undue reservation, to any qualified researcher.

## Ethics Statement

The studies involving human participants were reviewed and approved by Institutional Review Board, Colby College. Written informed consent from the participants' legal guardian/next of kin was not required to participate in this study in accordance with the national legislation and the institutional requirements.

## Author Contributions

GI conceptualized and designed the study. BM organized the database. JK performed the statistical analyses. GI, JK, and NC wrote sections of the manuscript. BM and PB helped design and coordinate data collection. RZ critically reviewed and edited the manuscript. PB wrote the IRB and conceptualized the overall project. All authors contributed to manuscript revision and read and approved the submitted version.

## Conflict of Interest

GI has been reimbursed by the government, professional scientific bodies, and commercial organizations for discussing or presenting research relating to MTBI and sport-related concussion at meetings, scientific conferences, and symposiums. He has a clinical practice in forensic neuropsychology, including expert testimony, involving individuals who have sustained mild TBIs (including athletes). He has received honorariums for serving on research panels that provide scientific peer review of programs. He is a co-investigator, collaborator, or consultant on grants relating to mild TBI funded by the federal government and other organizations. He has received research support from test publishing companies in the past, including ImPACT® Applications Systems, Psychological Assessment Resources, and CNS Vital Signs. He has received research support from the Harvard Integrated Program to Protect and Improve the Health of NFLPA Members, and a grant from the National Football League. He serves as a scientific advisor for BioDirection, Inc, Sway Medical, Inc., and Highmark, Inc. RZ has received salary support from the Harvard Integrated Program to Protect and Improve the Health of National Football League Players Association Members. He serves on the Scientific Advisory Board of Myomo, Oxeia Pharma, and ElMInda. The authors declare that this study received funding from the National Football League and ImPACT® Applications, Inc. The remaining authors declare that the research was conducted in the absence of any commercial or financial relationships that could be construed as a potential conflict of interest.
